# Pomegranate‐Inspired Graphene Parcel Enables High‐Performance Dendrite‐Free Lithium Metal Anodes

**DOI:** 10.1002/advs.202203178

**Published:** 2022-08-09

**Authors:** Long Zhang, Tao Ma, Yi‐Wen Yang, Yi‐Fei Liu, Peng‐Hu Zhou, Zhao Pan, Bi‐Cheng Hu, Chuan‐Xin He, Shu‐Hong Yu

**Affiliations:** ^1^ Department of Chemistry Institute of Biomimetic Materials & Chemistry Anhui Engineering Laboratory of Biomimetic Materials Division of Nanomaterials & Chemistry Hefei National Research Center for Physical Sciences at the Microscale Institute of Energy Hefei Comprehensive National Science Center University of Science and Technology of China Hefei 230026 P. R. China; ^2^ College of Chemistry and Environmental Engineering Shenzhen University Shenzhen 518060 P. R. China; ^3^ Institute of Innovative Materials Department of Materials Science and Engineering Department of Chemistry Southern University of Science and Technology Shenzhen 518055 P. R. China

**Keywords:** lithium metal anodes, bioinspired structural materials, graphene, lithium metal batteries, microrods

## Abstract

Uncontrolled lithium dendrites seriously hinder the commercialization of lithium metal batteries in comparison to the durable lithium‐ion batteries. Herein, inspired by squashy pomegranate structure, a novel loading strategy of metallic lithium (Li) is introduced to construct dendrite‐free Li metal anodes through porous reduced graphene oxide/Au (PRGO/Au) composite microrods (MRs) as unique storage parcels. The abundant internal voids and robust host structure are capable of achieving high mass loading of Li metal and effectively alleviating the conceivable volume change during cycling, accompanied by the preferential selective plating/stripping of Li inside the graphene‐based MRs with the embedded Au nanonuclei. As a result, the obtained PRGO/Au–Li anodes deliver a long‐lifespan stable cycling up to 600 h with a high specific capacity of ≈2140 mA h g^–1^ and voltage hysteresis as low as 20 mV in the absence of dendrites. The assembled full cells exhibit excellent rate capability and cycling stability. This work provides an alternative strategy to construct advanced high‐energy‐density lithium batteries via the unique 1D bioinspired graphene‐based packaging strategy.

## Graphical abstract

A unique storage parcel of porous reduced graphene oxide/ Au (PRGO/Au) is designed to construct dendrite‐free lithium metal anodes with high specific capacity. Owing to the embedded nanonuclei, abundant internal void, and robust host structure, the PRGO/Au–Li anodes deliver significantly improved electrochemical characteristics.

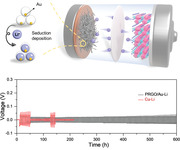

## 1 Introduction

With the ever‐increasing development of consumer electronics, electric vehicles, and grid‐scale energy storage, the demand for advanced electrode materials with higher energy density has become increasingly urgent.^[^ ^1^, ^2^, ^3^ ^]^ Among all kinds of anode materials, lithium (Li) metal, due to its high theoretical capacity (3860 mA h g^–1^) and most negative electrochemical potential (‐3.04 V vs standard hydrogen electrode), displays alluring prospect in the next‐generation high‐capacity rechargeable batteries.^[^ ^4^, ^5^ ^]^ However, owing to the inhomogeneous charge transport at the Li/electrolyte interface and infinite anode volume fluctuation during cycling, the solid electrolyte interphase (SEI) is usually more unstable in lithium metal batteries (LMBs), which inevitably induces the Li dendrite growth and continuous loss of electrolyte and active lithium.^[^ ^6^, ^7^, ^8^ ^]^ This further triggers the capacity decay and a suite of safety issues, and ultimately restricts its practical applications.^[^ ^9^, ^10^, ^11^ ^]^

To date, considerable efforts have been dedicated to the above‐mentioned challenges to improve the durability and safety of LMBs, and some fairly significant results have been achieved.^[^ ^12^, ^13^, ^14^ ^]^ These strategies can usually be summarized as engineering artificial SEI layers,^[^ ^15^, ^16^, ^17^ ^]^ modifying ingredients of electrolytes,^[^ ^18^, ^19^, ^20^ ^]^ and constructing 3D hosts for accommodating metallic lithium.^[^ ^21^, ^22^, ^23^ ^]^ As one of the most attractive strategies, the introduction of well‐designed 3D scaffolds can effectively alleviate the volume fluctuation and Li dendrite growth during plating/stripping via providing metallic Li storage space as well as homogenizing the interface current flow and Li‐ion flux.^[^ ^24^, ^25^, ^26^, ^27^ ^]^ Nevertheless, the concomitant large contact area derived from porous 3D hosts further intensifies the irreversible consumptions of metallic Li and electrolytes, leading to shorter cycling life and lower Coulombic efficiencies. Therefore, achieving stable cycling for LMBs remains a challenge.

Graphene, as an emerging monatomic layer carbon allotrope, has caught people's considerable attention to combine it into fabricating high‐performance LMBs owing to the excellent physicochemical properties.^[^ ^28^, ^29^, ^30^, ^31^, ^32^ ^]^ For example, wrinkled graphene cages studded with seeds as hosts for high‐capacity metallic Li anodes have greatly improved cycle lifetime.^[^ ^33^, ^34^ ^]^ Similarly, the reduced graphene oxide (RGO) film could provide a robust scaffold, excellent lithiophilicity, and stable artificial interface, which ultimately enabled uniform Li deposition and achieved a good rate capability for full cells.^[^ ^35^, ^36^, ^37^, ^38^ ^]^ Besides, the porous graphene framework could accommodate a larger amount of Lithium and maintain the stability of solid electrolyte interphase layer during Li plating/stripping.^[^ ^39^, ^40^, ^41^ ^]^ In our previous work,^[^ ^42^ ^]^ inspired by the vertical coiled channels in natural scallion stem as the highway for nutrients transport, a range of RGO‐based microrods were prepared and applied into LMBs. This unique scallion‐like wrapping design of graphene effectively improves rate performance and cycle stability in full battery due to the lower tortuosity and superior charge‐transfer kinetics. Moreover, other bioinspired structural materials were constructed to stabilize metallic Li anodes. For example, artificial wood‐like carbon host structure enabled lower polarization effect and better rate capability owing to low tortuosity derived from the vertical channels;^[^ ^43^ ^]^ the nacre‐like composite metallic Li anodes were designed by codepositing lithium with 2D vermiculite shuttles, which can absorb Li^+^, ferry it across a lithium substrate, and flatten the Li growth;^[^ ^44^ ^]^ bioinspired lotus root‐like 3D multichannel carbon‐based scaffolds can effectively guide the uniform deposition of lithium metal and mitigate the concomitant volume fluctuation.^[^ ^45^ ^]^ However, series of intrinsic issues such as complex synthesis process, low yields, high costs, or low specific capacities remain unavoidable, which ultimately hinders their commercial applications. Thus, it is of great significance to develop better strategies to stabilize metallic lithium anodes by the delicate assembly and utilization of graphene.

Herein, inspired by the multiscale package structure in natural squashy pomegranate, a kind of bioinspired porous graphene‐based microrods (PGMRs) was fabricated as unique 1D storage parcels to accommodate lithium metal and inhibit the dendrite growth. The robust conductive scaffold and abundant internal pore structure effectively increase the mass loading of Li metal, alleviating infinite volume change and concomitant structural stress. Besides, trace Au nanonuclei were introduced as seeds to guide the 3D spatially confined nucleation and subsequent plating of metallic lithium inside PGMRs. As a result, the as‐prepared porous reduced graphene oxide/Au–Li (PRGO/Au–Li) composite anodes demonstrated a high specific capacity of ≈2140 mA h g^–1^ and low voltage hysteresis of only 20 mV with an excellent reversible cycling up to 600 h in the absence of dendrites for symmetric cells. When matched with LiFePO_4_ cathodes, the full batteries with PRGO/Au–Li anodes delivered asignificantly improved rate capability and cycling stability relative to the contrast samples. In all, this work provides a simple but efficient strategy to simultaneously increase the mass loading of Li metal and suppress lithium dendrite growth via the unique 1D bioinspired graphene‐based packaging, which further enriches the application of graphene‐based macroscopic 1D nano‐assemblies in advanced high‐energy‐density lithium batteries.

## 2 Results and Discussion

### 2.1 Synthesis and Materials Characterization

**Figure 1:**
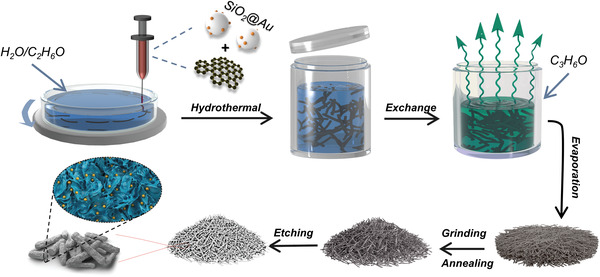
Schematic illustration of the preparation process of the PRGO/Au MRs.

The natural squashy pomegranate exhibits chamber characteristics, and every room contains seeds and sarcocarp inside. Inspired by this, combined with the intrinsically low tortuosity and high charge‐transfer kinetics derived from the scallion‐like graphene wrapping, we designed novel bioinspired porous graphene‐based microrods (PGMRs) as unique 1D storage parcels to accommodate lithium metal and inhibit the dendrite growth. The specific synthesis of the PGMRs was shown in **Figure** **1**. First, the silica nanoparticles with gold nanonuclei (SiO_2_@Au NPs) were prepared by the in situ aqueous‐phase reduction of soaking SiO_2_ NPs in dilute chloroauric acid. Subsequently, using graphene oxide (GO) and SiO_2_@Au NPs aqueous suspensions as injecting slurry, the reduced graphene oxide/Au (RGO/SiO_2_@Au) gel microrods (MRs) were obtained through our previously developed hydrothermal‐assisted wet‐spinning process.^[^ ^42^ ^]^ Further, a continuous solvent exchange, oven‐drying, grinding, and annealing process was successively applied into the preparation of RGO/SiO_2_@Au MRs. Ultimately, the corresponding porous reduced graphene oxide/Au (PRGO/Au) composite microrods (MRs) were synthesized by etching with hydrofluoric acid (HF).

**Figure 2:**
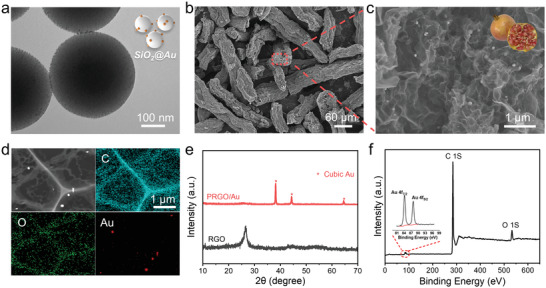
Characterization of the pomegranate‐like structure. a) TEM image of SiO_2_@Au NPs and conceptual graph insert. b) SEM image of prepared PRGO/Au MRs. c) The magnified SEM image of cross profile of PRGO/Au microrod. d) TEM image and EDS mapping of section of PRGO/Au microrod. e) XRD pattern of RGO/Au relative to pure RGO. (f) XPS spectra of PRGO/Au MRs.

The morphology of raw materials was characterized by transmission electron microscope (TEM). The results exhibited that the mean diameter of initial SiO_2_ NPs was ≈255 nm, and Au NPs (3–6 nm) were anchored onto the surface of silicon dioxide nanoparticles (Figures S1 and S2, Supporting Information and **Figure** **2**a). After assembly, SiO_2_@Au NPs were uniformly embedded into the scallion‐like graphene scaffolds as shown in Figure S3a–c (Supporting Information). Subsequently, silica nanotemplates were effectively dissolved and thoroughly removed by etching with HF solution, resulting in the ultimate PRGO/Au MRs. As shown in scanning electron microscope (SEM) and TEM (Figure 2b–d and Figure S3d–f, Supporting Information), the as‐prepared PRGO/Au MRs consist of the pomegranate‐like porous reduced graphene oxide (PRGO) hosts and sparsely embedded gold nanoparticles (Au NPs). The former provides abundant internal voids and flexible but robust interconnected conductive scaffold, which significantly increase the mass loading of Li metal and meanwhile ensure fast charge transport, along with effectively alleviating infinite volume fluctuation and concomitant structural stress. Compared to unannealed treatment, the number of the latter (Au NPs) in the product has significantly decreased and their size has obviously increased as observed by electron microscope and soft X‐ray tomography (Figure 2c,d and Figure S4a, Supporting Information), which may be attributed to the inevitable shedding on the fracture surface and the spontaneous fusion in adjacent cavities at high temperature. The corresponding X‐ray diffraction (XRD) and X‐ray photoelectron spectroscopy (XPS) patterns (Figure 2e,f) further confirmed that the nanoparticles in specimen are pure cubic Au, which is often considered as an effective nucleation seed to guide the confined plating/stripping of Li inside the specific 3D regions.^[^ ^25^, ^46^, ^47^ ^]^ Besides, it was found that the PRGO/Au MRs revealed a quite high Brunauer‐Emmett‐Teller (BET) surface area of 306 m^2^ g^–1^, demonstrating a plentiful porous structure for lithium storage inside MRs after removing silica nanotemplates as we expected (Figure S4b, Supporting Information).

### 2.2 Encapsulation of Lithium Metal

**Figure 3:**
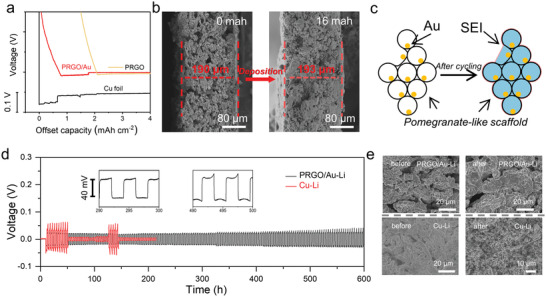
Electrochemistry Performance of PRGO/Au–Li electrode. a) Voltage–time curves during initial Li deposition at 0.02 mA cm^–2^ onto the Cu substrate and PRGO/Au electrode. b) Morphology change of the composite electrodes before and after Li deposition with mass loading of 16 mAh cm^–2^. c) The sketch map of lithium metal deposition process in PRGO/Au electrode. d) The symmetric battery cycle performance of PRGO/Au–Li and Cu/Li electrodes at 0.5 mA cm^–2^ with a capacity of 1 mA h cm^–2^. e) Surface morphology of Cu/Li and PRGO/Au–Li electrodes before and after 20 cycles.

As revealed in Figure 3a, the obtained PRGO/Au electrode exhibited a significantly lower overpotential than reference electrode, suggesting the preferential nucleation and subsequent directional deposition of metallic lithium within the PRGO/Au parcels.^[^ ^46^, ^48^, ^49^ ^]^ To verify the aforementioned anticipation, the morphology changes of the free‐standing PRGO/Au anodes in different stages of Li deposition were further investigated in detail. As shown in Figure 3b, after depositing 16 mA h cm^–2^ of lithium metal, the thickness of the composite electrode (5 mg cm^–2^ in weight) increases from 190 µm to 193 µm with a volume expansion of just 1.58%, which means a robust and stable porous lightweight framework for high‐capacity lithium storage (Table S1, Supporting Information). Meanwhile, the further SEM observations displayed that the pomegranate‐like cavity walls were first thickened and then the remaining cavity space was gradually filled to full during the Li plating process, resulting in a more compact and robust electrode structure (Figure S5, Supporting Information). As micro‐CT images shown in Figure S6 (Supporting Information), there were still plenty of gaps between MRs in the PRGO/Au–Li electrode with a high mass loading of 24 mA h cm^–2^ (a corresponding specific capacity of ≈2140 mA h g^–1^). All of these indicated that Li^+^ was indeed preferentially reduced at Au NPs and subsequently further packed inside the porous cavities of bio‐inspired PGMRs, which should be beneficial to effectively accommodating the high‐mass loading lithium metal, alleviating the structural stress originated from infinite volume fluctuation and suppressing Li dendrite growth during plating/stripping as illustrated in the schematic diagram (Figure 3c).^[^ ^24^, ^50^, ^51^ ^]^

To evaluate the integrated effects of the unique 1D bioinspired graphene‐based packaging, the corresponding symmetric cells were assembled and tested at different current densities for PRGO/Au‐Li and Cu/Li electrodes with a mass loading of 16 mA h cm^–2^. As shown in **Figure** **3**d, the obtained PRGO/Au‐Li composite anodes delivered a fairly smooth and stable voltage profile with a low overpotential of only 20 mV and excellent reversible cycling up to 600 h at 0.5 mA cm^–2^ for 1 mA h cm^–2^, while the contrast Cu/Li samples rapidly failed after only nearly 50 h due to the internal short circuit stemming from the uncontrolled Li dendrite with an obviously larger nucleation overpotential. In addition, the symmetric cell tests at other current densities further confirmed that the electrochemical characteristics of PRGO/Au–Li electrodes were significantly superior than these of the reference samples (Figure S7, Supporting Information). After 20 cycles, the surface of the disassembled PRGO/Au‐Li electrode remained smooth and dendrite‐free, while the Cu–Li anode revealed distinctly dendritic filament morphology (Figure 3e). These results mean that the constructed bio‐inspired PRGO/Au parcels could effectively accommodate and guide the 3D spatially selective deposition of Li with stabilizing solid electrolyte interphase layer and suppressing lithium dendrite formation during plating/stripping as we expected.^[^ ^23^, ^46^, ^52^ ^]^

**Figure 4:**
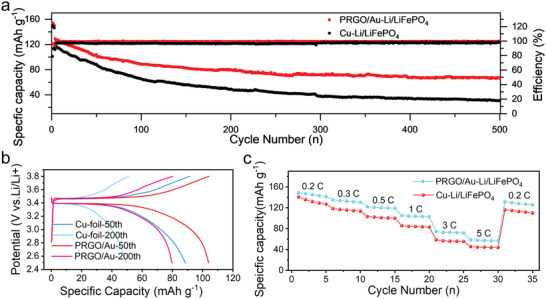
Electrochemical performance of PRGO/Au–Li/LiFePO_4_ full cells. a) Long‐term cycling tests of full batteries based on PRGO/Au–Li and Cu–Li anodes matched with LiFePO_4_ (LFP) as cathodes at 0.5 C. b) Typical charge and discharge curves of PRGO/Au–Li/LiFePO_4_ full batteries at 0.5 C for different cycles. c) Rate capability of PRGO/Au–Li/LiFePO_4_ full cells.

To further verify the practical potential of the resultant squashy pomegranate structure of PRGO/Au–Li electrodes with a mass loading of 16 mA h cm^–2^, full cells were assembled with LiFePO_4_ slices as cathodes (mass loading ≈2 mg cm^–2^). As depicted in **Figure** **4**a, the obtained PRGO/Au–Li/LiFePO_4_ full battery exhibited a obviously superior cycling stability and higher discharge specific capacity to that of the Cu–Li‐based counterpart at 0.5 C. In this stage, the PRGO/Au–Li battery delivered a specific capacity of up to 129 mA h g^–1^, which was significantly higher than that of the reference cell (119 mA h g^–1^). After 500 cycles, the capacity of PRGO/Au–Li/LiFePO_4_ full cells still retained a specific capacity of 62 mA h g^–1^ with a capacity decay as low as 0.1% per cycle (vs ≈ 29 mA h g^–1^ for Cu–Li/LiFePO_4_ battery). Interestingly, the corresponding charge‐discharge profiles exhibited that the PRGO/Au‐Li/LiFePO_4_ full battery brought much lower voltage polarization with a nearly 100% coulombic efficiency during 500 cycles. Furthermore, PRGO/Au‐Li/LiFePO_4_ battery delivered greatly improved rate capability than that of the reference sample as shown in Figure 4c. All this means that the unique 1D bioinspired graphene‐based packaging leads to a more stable metallic Li‐based anode and a better charge‐transfer kinetics during cycling.

## 3 Conclusion

In summary, inspired by squashy pomegranate structure, we developed a novel bioinspired graphene‐based packaging strategy to construct dendrite‐free metallic Li‐based anodes. The robust conductive scaffold and abundant internal voids provided abundant storage space to accommodate lithium metal, and mitigated infinite volume fluctuation and the concomitant structural stress, which significantly increased the mass loading for Li metal and effectively stabilized solid electrolyte interphase layer during cycling. Besides, the introduction of trace Au nanonuclei enabled the 3D spatially preferential selective plating/stripping of Li inside the graphene‐based MRs. In this context, the resultant PRGO/Au–Li composite electrodes displayed a long‐lifespan stable cycling up to 600 h with a low hysteresis at 20 mV and a high specific capacity of 2140 mA h g^–1^ in absence of dendrites. When coupled with LiFePO_4_ cathodes, the assembled PRGO/Au–Li/LiFePO_4_ full cells delivered significantly improved rate capability and cycling stability with much lower voltage polarization with a nearly 100% coulombic efficiency than that of the reference sample. Thus, this work provides a new bioinspired strategy to construct advanced high‐energy‐density and high‐stability lithium metal anodes.

## 4 Experimental Section

### Materials

The graphene oxide solution was prepared by the modified Hummers’ method.^[^ ^19^, ^42^ ^]^ SiO_2_ nanoparticles were synthesized following the previous publication.^[^ ^53^ ^]^ LiFePO_4_ powder was purchased for Guangdong Canrd New Energy Technology Co., Ltd. Muti‐walled carbon nanotubes (MWCNTs) were bought from Suzhou Tanfeng Graphene Technology Co., Ltd. Lithium foils were bought from China Energy Lithium Co., Ltd. Other chemicals were purchased from Shanghai Aladdin Bio‐Chem Technology Co., Ltd. All the chemicals were used as received without any purification.

### Preparation of SiO_2_@Au Core–Shell Nanoparticles (NPs)

The core–shell structure was realized by the modified method from the previous publication.^[^ ^54^ ^]^ In a typical synthesis, first, the pH of 10 mL of HAuCl_4_ solution (0.1 m) was adjusted to neutral by dropwise addition of 1 m aqueous NaOH solution. And then, 0.5 g of silica nano‐spheres (≈255 nm) were added into the above‐mentioned solution with vigorous ultrasonic agitation. After 5 minutes, 20 mL of ethanol was added and heated at 96 °C for 15 min. Subsequently, the SiO_2_@Au NPs were collected by five centrifugal washing with deionized water at 1000 rpm. Finally, the suspension A was prepared by reultrasonically dispersing the above precipitate into 5 mL of deionized water for later use.

### Preparation of RGO/SiO_2_@Au Microrods (MRs)

First, the injection slurry was obtained by mixing 32.5 mL of GO aqueous solution (7.8 mg mL^‐1^) with the suspension A. Subsequently, the mixed slurry was injected into the rotating CaCl_2_‐based coagulation bath (5 g of CaCl_2,_ 200 mL of H_2_O and 300 mL of ethanol). The collected GO/SiO_2_@Au MRs‐based mixture was poured into the stainless‐steel autoclave, heated in an oven at 160 °C for 12 h, and then naturally cooled to room temperature. The resultant RGO/SiO_2_@Au gel MRs suspensions were successively washed with deionized water and acetone followed by a fast oven‐drying at 90 °C. Finally, the RGO/SiO_2_@Au MRs were obtained by a continuous grinding in a household mini coffee grinder for 10 s and subsequent annealing treatment at 600 °C for 2 h in the argon atmosphere.

### Preparation of the Pomegranate‐Like PRGO/Au MRs

The RGO/SiO_2_@Au MRs powder were cast into the HF etching solution (HF:H_2_O:EtOH = 10:30:5). The suspension was magnetically stirred at room temperature for 12 h, and then mixture was washed and filtered. Subsequently, the above process was repeated again. Ultimately, the desired product was acquired by further oven drying.

### Characterization

Scanning electron microscopy (SEM) was carried out using a GeminiSEM 500 at an acceleration voltage of 5 kV. Transmission electron microscopy (TEM) images were obtained with Hitachi H‐7650 at an acceleration voltage of 120 kV. X‐ray power diffraction (XRD) patterns were recorded on a Philips X'Pert X‐ray diffractometer with Cu K*α* radiation. X‐ray photoelectron spectra (XPS) for PRGO/Au MRs were determined by an X‐ray photoelectron spectrometer (ESCA Lab MKII) with Mg K*α* radiation. TEM images and EDS maps were obtained with JEOL‐2100F microscope at an acceleration voltage of 200 kV after slicing a PRGO/Au microrod in 200 nm slices, which are prepared in Core Facility center for Life Sciences. Soft X‐ray tomography were performed with the soft X‐ray microscopy at BL07W beamline in the National Synchrotron Radiation Laboratory (NSRL). X‐ray CT was tested with Xradia 520 versa in USTC Center for Micro‐ and Nanoscale Research and Fabrication.

### Electrochemical Measurements

PRGO/Au–Li anodes were fabricated by a conventional doctor blade‐casting method. In a typical procedure, a certain amount of PRGO/Au MRs powders, polyvinylidene fluoride (PVDF), and carbon nanotubes with a mass ratio of 8:1:1 were dispersed in N‐methyl‐2‐pyrrolidone (NMP) and stirred overnight. The obtained mixture was then scraped onto copper foils and dried at 110 °C in a vacuum oven for 12 h. Subsequently, the as‐prepared electrode was cut into 1.13 cm^–2^ circular disks with mass loading of ≈5 mg. Finally, all PRGO/Au‐based disks were transferred into an Ar‐filled glove box with water and oxygen contents < 0.1 ppm, and assembled into 2032 cells with an ether electrolyte (1.0 m LiTFSI, 5% LiNO_3_ in 1,3‐dioxolane/1,2‐dimethoxyethane (DME:DOL = 1:1 (volume ratio)) for the relevant tests.

All cell measurements were carried out using a NEWARE multichannel electrochemical testing system.

## Conflict of Interest

The authors declare no conflict of interest.

## Supporting information

Supporting Information

## Data Availability

The data that support the findings of this study are available from the corresponding author upon reasonable request.
