# Supplementary material for: Pomegranate‐Inspired Graphene Parcel Enables High‐Performance Dendrite‐Free Lithium Metal Anodes
Source: Adv Sci (Weinh). 2022 Aug 9;9(28):2203178. doi: 10.1002/advs.202203178 (PMC9534963; doi:10.1002/advs.202203178)
Supplement: Supplementary file 1 — Supporting Information [file ADVS-9-2203178-s001.pdf]

ADVANCED SCIENCE
Open Access

Supporting Information

for *Adv. Sci.*, DOI 10.1002/advs.202203178

Pomegranate-Inspired Graphene Parcel Enables High-Performance Dendrite-Free Lithium Metal Anodes

*Long Zhang, Tao Ma, Yi-Wen Yang, Yi-Fei Liu, Peng-Hu Zhou, Zhao Pan, Bi-Cheng Hu, Chuan-Xin He and Shu-Hong Yu**

Supporting Information

Pomegranate-inspired graphene parcel enables high-performance dendrite-free lithium metal anodes

*Long Zhang, Tao Ma, Yi-Wen Yang, Yi-Fei Liu, Peng-Hu Zhou, Zhao Pan, Bi-Cheng Hu, Chuan-Xin He and Shu-Hong Yu**

†These authors contributed equally to this work.

*Correspondence to: shyu@ustc.edu.cn.

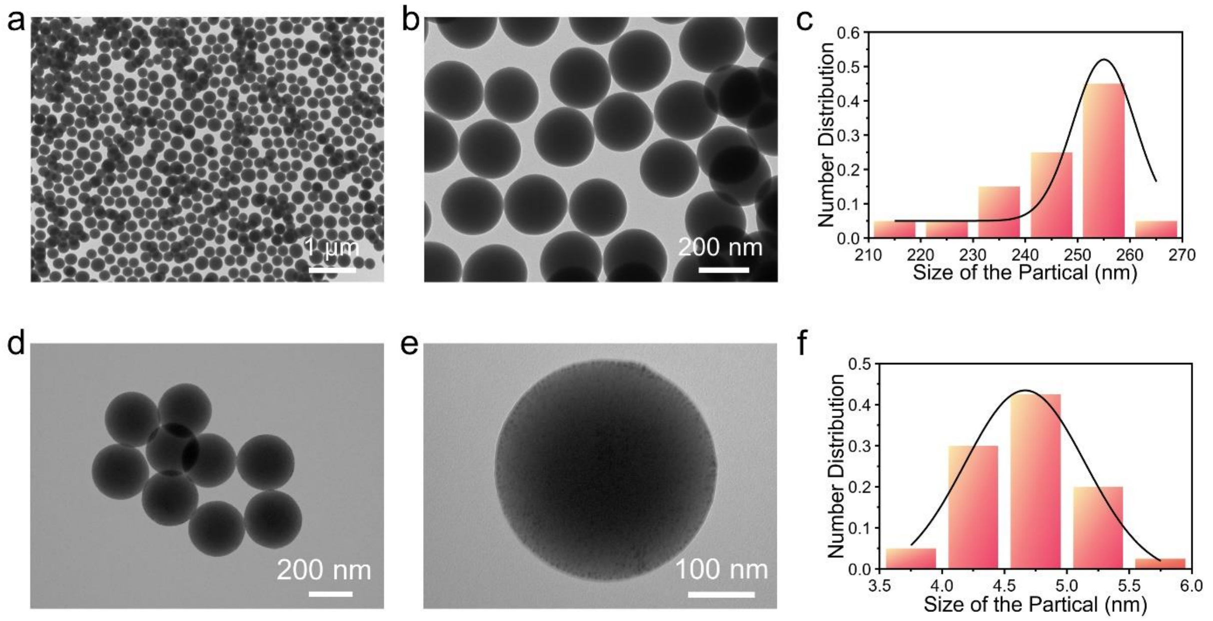

Figure S1. (a-b) and (d-e) SEM images of SiO_2 and SiO_2@Au nanoparticles (NPs). **(c)** and **(e)** Size distribution of SiO_2 and SiO_2@Au NPs, respectively.

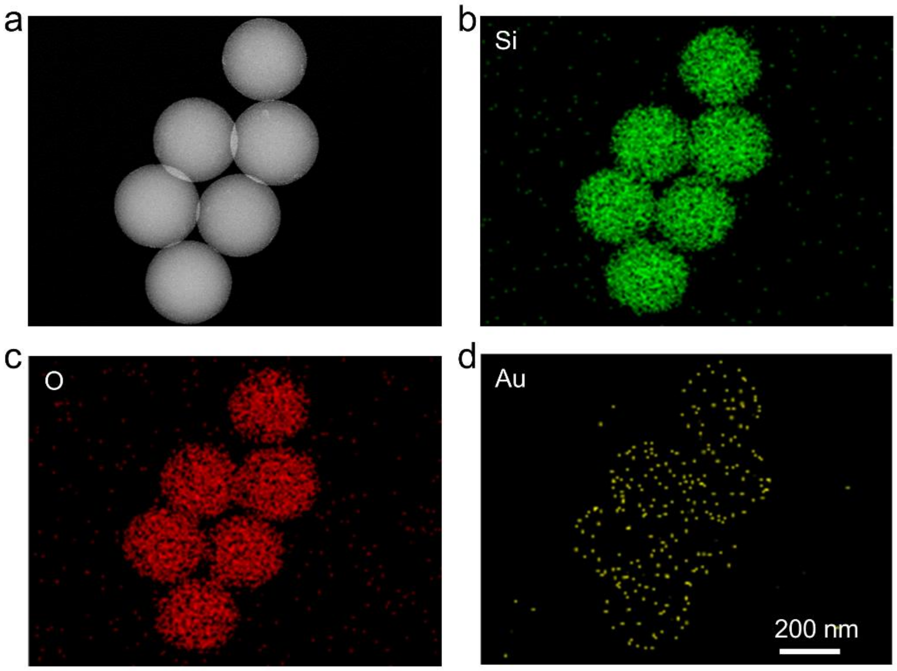

Figure S2. TEM image and corresponding element maps of SiO_2@Au NPs.

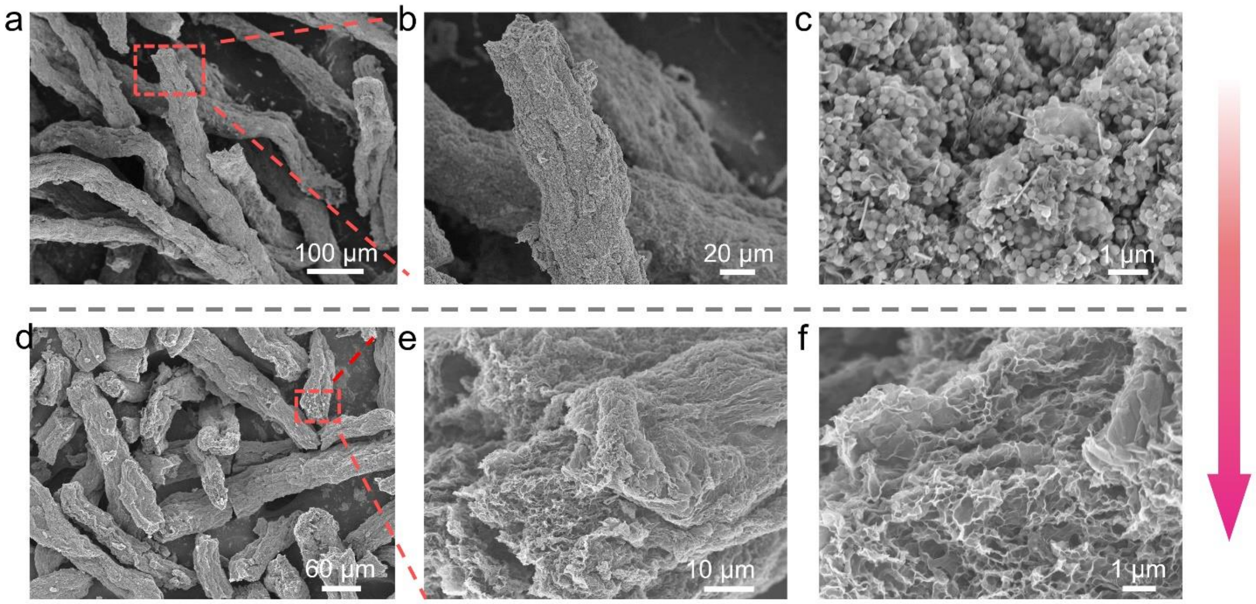

Figure S3. (a,c), The SEM images of the as-prepared RGO/SiO_2@Au micro-rods (MRs). **(d,f)** The SEM images of porous reduced graphene oxide/Au (PRGO/Au) composite MRs.

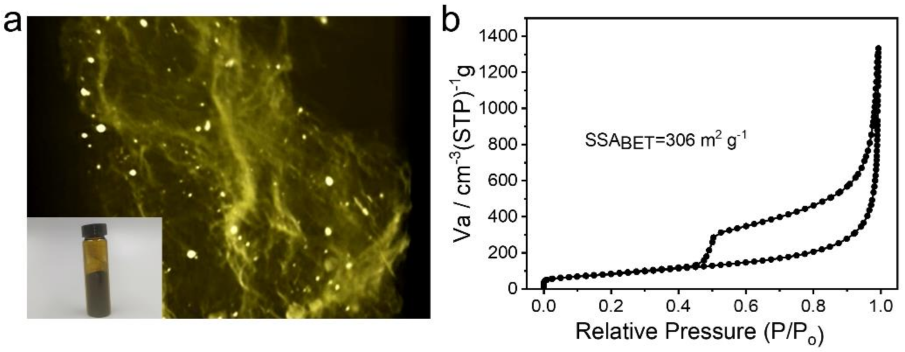

Figure S4. (**a**) Soft X-ray Tomography of the corresponding PRGO/Au-based fragment and optical image for PRGO/Au MRs powder (**b**) N_2 adsorption-desorption isotherms of PRGO/Au MRs.

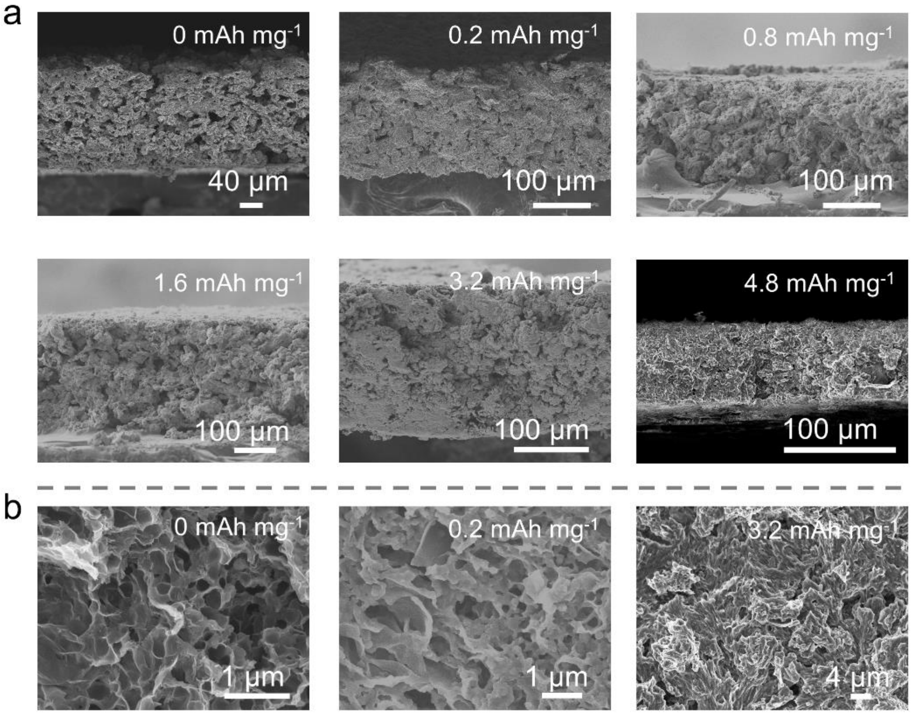

Figure S5. (**a**) Cross-sectional SEM images of PRGO/Au-Li electrodes with different mass loading. (**b**) Magnified SEM images, which showed the clear morphology transformation of PRGO/Au-Li electrodes at various discharging state.

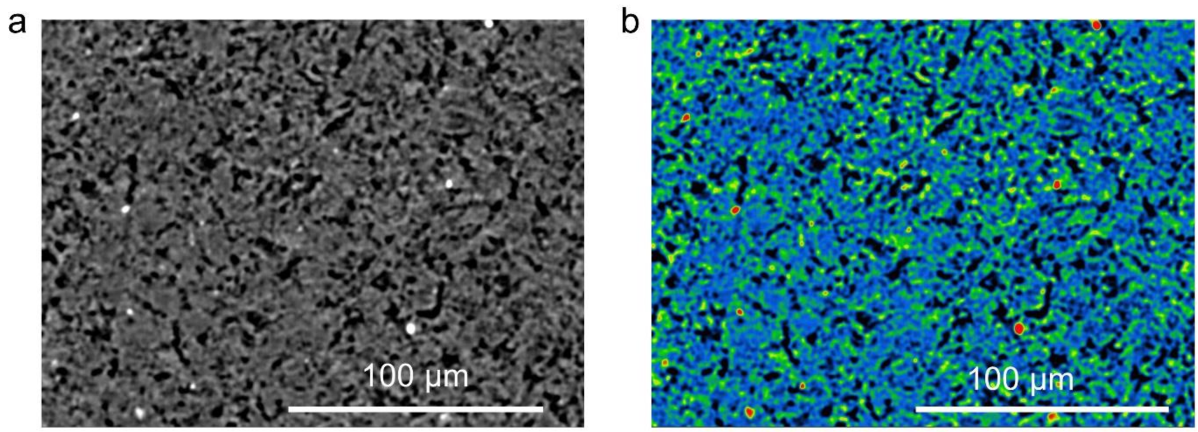

Figure S6. (a) The X-ray CT result of PRGO/Au-Li electrode with mass loading of 24 mA h cm^{-2}. **(b**) The homologous simulated diagram.

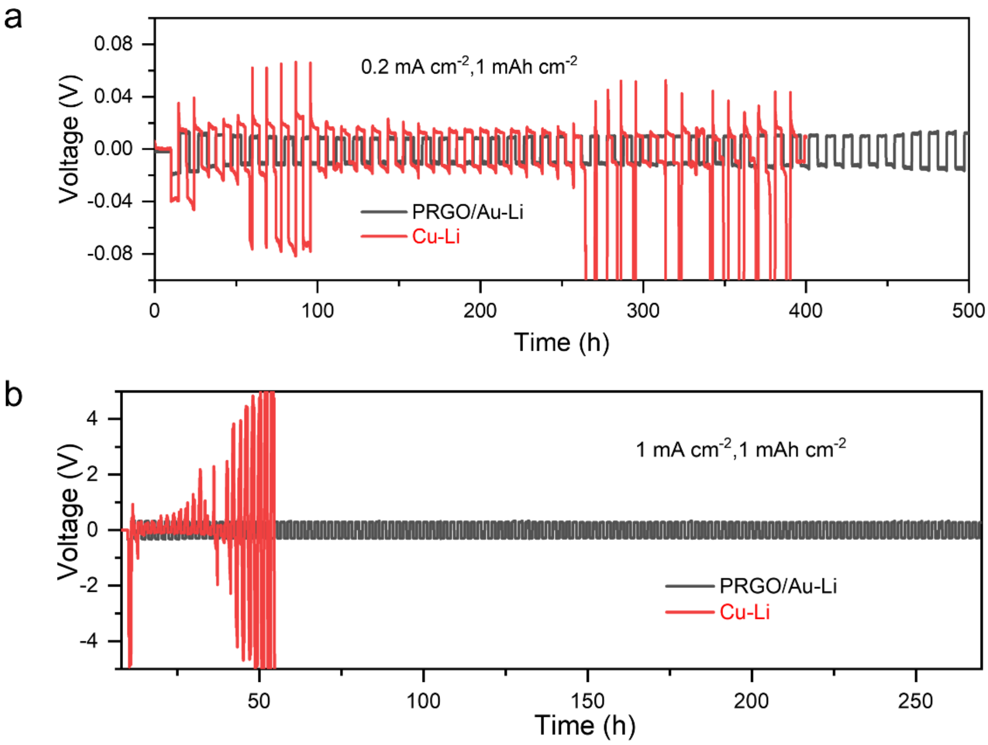

Figure S7. The cycle performance at 0.2 mA cm^{-2} (a)and1 mA cm^{-2} (b) with the mass loading of 16 mA h cm^{-2} in symmetric batteries.

Table S1. Scaffolds for Lithium metal anodes.

Scaffold	Thickness expansion rate	Capacity (mA h cm^{-2})	Specific Li storage capacity (mA h g^{-1})	Full Cells			
				Cathode type	Rates or Current density	Decay rate of each cycle (%)	cycles
Crumpled graphene ball[1]	9.2%	10	-	LFP	0.5C	-	-
Au-RGO[2]	33%	2	-	-	-	-	-
Hierarchical silver-nanowire–graphene host[3]	-	22	-	NCM523	10C	0.062%	1000
3D graphene framework[4]	10%	4	-	LFP	0.5C	0.05%	200
ZnO nanoparticle (NP) confined 3D porous carbon[5]	10%	2	-	LFP	1C	0.02%	300
3D multichannel carbon fibers[6]	11.1%	-	2987.6	LFP	1C	0.014%	600
Hard carbon[7]	125%	4	-	NCM622	0.3C	0.21%	160
Hollow porous multi-nanochannel carbon fiber[8]	1.6%	5	-	LFP	0.5C	0.03%	400
3D porous biochar modified by silver nanoparticles[9]	43%	-	2474	-	-	-	-
3D graphene hosts[10]	9.5%and 12.6%	1 and 2	-	NCM	1 A g-1	0.036%	600
This work	1.58%	16	2140	LFP	1C	0.1%	500

References

[1] S. Liu, A. Wang, Q. Li, J. Wu, K. Chiou, J. Huang, J. Luo, Joule **2018**, 2, 184.

[2] J. Pu, J. Li, Z. Shen, C. Zhong, J. Liu, H. Ma, J. Zhu, H. Zhang, P. V. Braun, Adv. Funct. Mater. **2018**, 28, 1804133.

[3] P. Xue, S. Liu, X. Shi, C. Sun, C. Lai, Y. Zhou, D. Sui, Y. Chen, J. Liang, Adv. Mater. **2018**, 30, e1804165.

[4] L. Pan, Z. Luo, Y. Zhang, W. Chen, Z. Zhao, Y. Li, J. Wan, D. Yu, H. He, D. Wang, ACS Appl. Mater. Interfaces **2019**, 11, 44383.

[5] L. Tang, R. Zhang, X. Zhang, N. Zhao, C. Shi, E. Liu, L. Ma, J. Luo, C. He, J. Mater. Chem. A **2019**, 7, 19442.

[6] L. Yu, Q. Su, B. Li, W. Liu, M. Zhang, S. Ding, G. Du, B. Xu, Electrochim. Acta **2020**, 362, 137130.

[7] Y. Liu, X. Wu, C. Niu, W. Xu, X. Cao, J.-G. Zhang, X. Jiang, J. Xiao, J. Yang, M. S. Whittingham, J. Liu, ACS Energy Lett. **2021**, 6, 1550.

[8] J. Zhang, Z. Su, J. Jin, S. Yang, A. Yu, G. Li, ACS Appl. Mater. Interfaces **2021**, 13, 39311.

[9] T. Zhao, M. Wang, Y. Yao, F. Yang, Y. Jiang, D. Mu, F. Wu, C. Zhang, Electrochim. Acta **2021**, 388, 138632.

[10] Y. H. Kim, G. W. Lee, Y. J. Choi, H. S. Choi, K. B. Kim, Adv. Funct. Mater. **2022**, 2113316. , https://doi.org/10.1002/adfm.202113316
